# Reactive surveillance and response strategies for malaria elimination in Myanmar: a literature review

**DOI:** 10.1186/s12936-023-04567-6

**Published:** 2023-04-27

**Authors:** Win Htike, Nay Yi Yi Linn, Kyawt Mon Win, Lae Shwe Sin Myint, May Chan Oo, Ei Phyu Htwe, Aung Khine Zaw, Katherine O’Flaherty, Paul A. Agius, Ellen A. Kearney, Freya J. I. Fowkes, Win Han Oo

**Affiliations:** 1 Health Security and Malaria Program, Burnet Institute Myanmar, Yangon, Myanmar; 2 Faculty of Health, School of Medicine, Deakin University, Melbourne, VIC Australia; 3 Department of Public Health, Ministry of Health, Nay Pyi Taw, Myanmar; 4 Disease Elimination Program, Burnet Institute, 85 Commercial Road, 3004 Melbourne, VIC Australia; 5 Biostatistics Unit, Faculty of Health, Deakin University, Melbourne, VIC Australia; 6 Department of Epidemiology and Preventive Medicine, Monash University, Melbourne, VIC Australia; 7 Melbourne School of Population and Global Health, University of Melbourne, Melbourne, VIC Australia

**Keywords:** 1-3-7 strategy, Malaria elimination, Reactive surveillance and response

## Abstract

Myanmar, a country in Greater Mekong Sub-region, aims to eliminate malaria by 2030. To achieve malaria elimination, Myanmar adopted a reactive surveillance and response strategy of malaria case notification within 1 day and case investigation, foci investigation and response activities within 7 days. A literature review was conducted to gain a better understanding of how the reactive surveillance and response strategies are being implemented in Myanmar including enablers and barriers to their implementation. Only two assessments of the completeness and timeliness of reactive surveillance and response strategy in Myanmar have been published to date. The proportion of positive cases notified within one day was 27.9% and the proportion of positive cases investigated within 7 days as recommended by the national guidelines varied from 32.5 to 91.8% under different settings in reported studies. Strong collaboration between the National Malaria Control Programme and implementing partners, and adequate human resource and financial support contributed to a successful and timely implementation of reactive surveillance and response strategy. Documented enablers for successful implementation of reactive surveillance and response strategy included frontline health workers having good knowledge of reactive surveillance and response activities and availability of Basic Health Staff for timely implementation of foci response activities. Barriers for implementation of reactive surveillance and response activities were also identified, including shortage of human resources especially in hard-to-reach settings, limited mobile phone network services and internet coverage leading to delays in timely notification of malaria cases, lengthy and complex case investigation forms and different reporting systems between Basic Health Staff and volunteers.

**Supplementary Information:**

The online version contains supplementary material available at 10.1186/s12936-023-04567-6.

## Background

Myanmar, a country in the Greater Mekong Sub-region (GMS), has made significant reductions in its malaria burden over the last decade. Between 2010 and 2020, the number of malaria cases in Myanmar was reduced by 90%, the number of malaria deaths by 99% and the number of indigenous malaria cases by 86% [1].

Despite a successful control effort, Myanmar is threatened by the emergence of artemisinin-resistant malaria in the GMS [2–4]. To tackle the problem of artemisinin resistance [5], the countries of the GMS, including Myanmar, committed to eliminating malaria by 2030 [6]. Strengthening malaria surveillance is fundamental to malaria programme planning and implementation, and is crucial for accelerating progress towards elimination [7]. Once a country is aiming for malaria elimination, it should “*enhance surveillance to ensure that every infection is detected and implement targeted measures for attacking both parasites and vectors in order to interrupt local transmission, eliminate all parasites from humans, and manage the risk of re-establishment through imported malaria*” [7]. Once a parasitologically-confirmed malaria case is detected by a health worker or a volunteer, timely case notification, case investigation and focused investigation must be conducted for reliable determination of the source of infection and classification of cases and foci to inform an appropriate response. These activities are interconnected and are referred to as case detection and notification, case investigation and classification, and foci investigation and response, or herein collectively referred to as reactive surveillance and response (RASR) activities [8].

Since the National Programmes of the GMS countries are undergoing programme reorientation from control to elimination, it is vital to evaluate the performance and feasibility of the implementation of RASR strategies in order to provide recommendations for improved RASR in terms of quality, effectiveness, and coverage in the context to existing national health systems which will contribute to achieving malaria elimination goals in the GMS. This literature review is part of the multi-country assessment of RASR strategies in the GMS countries, and aims to comprehensively review and analyse the RASR strategies that are currently used in Myanmar with specific objectives of (1) assessing context-specific and timely implementation of RASR strategies in Myanmar, and (2) documenting barriers and enablers for successful implementation of RASR strategies in Myanmar.

### Search strategy

A literature search was undertaken in the PubMed database using the search terms “Myanmar”, “surveillance and response” and “malaria elimination” in title and abstract {PubMed search term: [(myanmar [Title/Abstract]) AND (surveillance [Title/Abstract] AND response [Title/Abstract])] AND (malaria elimination [Title/Abstract])}. Articles that reported the completeness and timeliness of RASR activities, and those that reported the barriers and enablers of implementing RASR activities in Myanmar were selected and one additional article was extracted from the reference list. Grey literature such as national strategies and guidelines on RASR strategies in Myanmar and sub-national data on completeness of RASR activities in Myanmar extracted from presentations of the Annual Review Meeting of Myanmar Vector Borne Diseases Control Program conducted on 20–22 July 2022 in Nay Pyi Taw, Myanmar was also included in this review.

## Results

### Overall policy and guidelines on reactive surveillance and response in Myanmar

To achieve the goal of malaria elimination in Myanmar by 2030, the National Malaria Control Programme (NMCP) started implementing a RASR strategy of “1–7” in 2016 based upon the “1-3-7” surveillance and response strategy [9]. The “1-3-7” strategy is a malaria surveillance strategy launched by China in 2012, which refers to case notification within 1 day, case investigation and classification within 3 days, and foci investigation and responses within seven days [10]. Myanmar NMCP adopted it by combining “3” and “7” components of the original “1-3-7” strategy into “7”. In August 2020, the NMCP also developed the “Malaria Elimination Field Implementation Manual (MEFIM)” which describes the standard operating procedures for malaria elimination activities, including RASR, to be executed by all the implementing partners in Myanmar. The major RASR activities include case notification, case investigation, and foci investigation and responses [11]. In addition, the NMCP recommends all implementing partners conduct preparatory activities before implementing the RASR activities including – development of village-based stratification; development of reporting forms, formats, and registers; training of health staff; strengthening routine case detection and treatment activities; establishment of District/Township Malaria Elimination Management Team which is led by District/Township Medical Officer and comprises of township level Basic Health Staff (Health Assistant, Midwife, Lady Health Visitor, Public Health Supervisors), Vector Borne Disease Control Programme (VBDC) focal person and implementing partners as well as health staff from sub-township level; establishment of Township Malaria Elimination Coordination Committee; organizing advocacy meetings at township level; and launching malaria elimination activities at State/Regional level [11].

### Case notification

As per the policy, all malaria cases must be notified to the designated focal person at the township level within 24 h of diagnosis [11]. Case notification can be done by direct phone calling, sending short messaging service, using the Malaria Case-based Reporting and Surveillance (MCBRS) application (an android-based mobile phone application developed by NMCP, Save the Children, and WHO for malaria reporting) or in-person using the standardized case notification form (Additional file 1).

Upon being notified of a case, the designated focal person at township level may provide initial instructions for treatment as per the national treatment guideline if needed and homecare management including family directly observed treatment. The designated focal person also needs to inform the malaria case to the township Malaria Elimination Management Team, which will allot case identification number, record in the positive case register, and prepare and schedule for case investigation, foci investigation and response activities [11].

### Case investigation

The Myanmar NMCP modified “1-3-7” strategy into “1–7” to reflect the country’s situation and capacity. The NMCP does not specify different timelines for “3” and “7” parts of the “1-3-7” strategy (i.e., case investigation and foci investigation and response activities). Instead, it recommends to do both the case investigation, and foci investigation and response activities as soon as possible but not later than 7 days after the case is diagnosed [6, 11] (Table 1).

**Table 1:** Summary of reactive surveillance and response (RASR) strategies for malaria elimination in Myanmar [11]

Type of malaria RASR activity	Time schedule	Key implementers (Under the guidance of District and Township Medical Officer)	Expected outcome
Case notification	Within 24 h of diagnosis	Village Malaria Worker, Public Health Supervisor − 1 and 2, midwife, Malaria supervisor, permanent spray-man and field staff from the implementing partners	Front line health worker reports malaria cases to township focal person within 24 h of diagnosis
Case investigation	Within 7 days of diagnosis	Township Health Assistant / Health Assistant 1, Malaria Assistant, Lady Health Visitor, Midwife, Malaria Inspector, Public Health Supervisor − 1 and 2, and field staff from the implementing partners, Malaria Supervisor, Permanent Spray-man, Village Malaria Worker	Case classified as indigenous, introduced, imported, relapse/ recrudescent, induced, or cryptic
Foci investigation			Foci classified as active, residual non-active, or cleared
Foci response			Focus received appropriate response activities

After a malaria case is notified to the designated township focal person, the township Malaria Elimination Management Team is required to visit the village of the notified malaria case to conduct case investigation, foci investigation and appropriate response activities (Table 1). Case investigation is done by a trained health staff (e.g., Health Assistant, Midwife, VBDC staff) using a standardized case investigation form (Additional file 2). During a case investigation, the health staff needs to meet the notified index malaria cases and collect their sociodemographic information, travel history, data on recent treatment for malaria, undertake an assessment of the possibility of onward malaria transmission from the index malaria case, and develop a summary report of the case investigation [11]. Following case investigation, the notified malaria case will be classified as per WHO guidelines [12] either as “indigenous”, “introduced”, “imported”, “relapse/recrudescent”, “induced”, or “cryptic” by the investigator, which will sequentially be checked and approved by the respective State/Regional Malaria Officer (Additional file 3). The result of case classification will also support the subsequent classification of the foci [11].

### Foci investigation and response

After the notified case is investigated and classified, the foci investigation team starts to investigate the focus, “*a geographically well-defined and circumscribed area situated in a currently or formerly malarious area that contains the epidemiological and ecological factors necessary for malaria transmission*” [13], using a standardized foci investigation form (Additional file 4). A foci investigation team usually comprises malaria focal persons from the regional and township VBDC team and Basic Health Staff from the respective area (Table 1) and collects information on receptivity and vulnerability of the area to malaria (Additional file 4). Such information includes climatic conditions, abundance of mosquitoes, topography of the village, housing quality, usage of ITN/LLIN and IRS, possibility of environmental manipulation or modification for larval source management such as clearing vegetation, proper drainage and flow of water, landfilling of small shallow unused wells at the side of streams nearby the human dwellers, permanent drainage of water flow, presence of night-time human activities, estimated number of non-immune population (people with no history of malaria during the past 1 year) among villagers, vulnerability to malaria such as influx of individuals or populations from a malaria endemic area and importation of malaria vector, quality of available health care services, and treatment-seeking behaviour of the population [11]. Afterwards, the focus is classified either as “active” (areas with ongoing transmission), “residual non-active” (areas with recent local malaria transmission within 1–3 years), or “cleared” (areas where transmission has been interrupted for more than 3 years) by the investigation team and approved by the State/Regional Malaria Officer [11].

Response activities are conducted according to the findings from case and foci investigation and classification. Response activities vary according to the type of focus and include, but are not limited to, passive, active and reactive case detection; vector control measures including distribution of LLIN and IRS; and timely reporting and notification of malaria cases (Table 2). In addition, it is recommended to do community awareness raising; regular larva source management; training and retraining of Integrated Community Malaria Volunteers (community-based malaria volunteers to provide malaria prevention, diagnosis, case management, and referral services integrated by activities for dengue, lymphatic filariasis, tuberculosis, HIV/AIDS, and leprosy) on case detection and treatment; and assessment of surveillance and health system [11].

**Table 2:** Case detection and vector control activities based on foci classification results [11]

Type of foci	Recommended case detection and vector control measures
Active foci in high transmission area	• Regular passive case detection throughout the year • Regular active case detection every three months especially during transmission season with high malaria caseload • Additional reactive case detection if positive case is found • Expansion of focus area and mass screening if additional positive cases are detected during reactive case detection • Indoor residual spraying for three consecutive years
Active foci in moderate to low transmission area	• Regular passive case detection throughout the year *plus* active case detection during transmission season • Reactive case detection in foci area by testing all suspected malaria cases • Distribution of LLIN to all households to get the universal coverage
Residual non-active foci (If receptivity is present and transmission was present past 1–2 years ago)	• Regular passive case detection throughout the year *plus* active case detection during transmission season or if mobile and migrant population are present • Reactive case detection to all co-travellers and people having malaria risk • Testing of family members and neighbours if the household of positive case is receptive (i.e., presence of possible breeding places) and the positive case is detected late • Distribution of LLIN to residents and migrants based on receptivity and vulnerability status • Reclassification of foci and assessment of surveillance system if a locally contracted malaria case is detected and taking key immediate responses including mass screening and treatment, indoor residual spraying, and strengthening the surveillance system
Cleared foci	• Regular passive case detection throughout the year *plus* weekly regular active case detection if the area is receptive and migrant population are present • Testing of all suspected malaria cases if a non-locally contracted malaria case is detected • Mass screening and treatment, distribution of personal protective measures and self-notification of fever cases if there is a possibility of re-introduction of cases • Compulsory reporting and testing of all visitors or migrant workers from other places regardless of fever • Surveillance system strengthening

After conducting field activities, the reporting forms and formats of RASR must be stored properly and it is recommended to submit the activity report on RASR for each index case to the State/Regional Malaria Officer within 3 days.

### Experiences in the field implementation of reactive surveillance and response activities

Although Myanmar has MEFIM guidelines, standard operating procedures, and guidelines for RASR, actual field implementation of RASR were somewhat different. Generally, case notification and investigation activities were conducted according to the MEFIM guidelines. After being notified, the township level staff (either Basic Health Staff or VBDC focal person) undertook the case investigation and classification using the standardized forms (Additional files 2 and 3). The positive case was also recorded in the positive case register (Additional file 5) which was kept at the Regional VBDC office (Regional Malaria Assistant, personal communication on 10th Nov 2022).

The Regional VBDC team arranged to conduct foci investigation and response activities afterwards, if no such activities had been conducted within the last calendar year in that area. According to the MEFIM guidelines, foci investigation and response activities are supposed to be conducted by the township or district level Malaria Elimination Management Team, however, the Malaria Elimination Management Teams are yet to be established in most areas, leaving the Regional VBDC is responsible for all the foci investigation and response activities. After the team had conducted foci investigation and response activities, it was reported to the Regional Malaria Officer using the standardized form (Additional file 4). The outcomes of foci investigation and response were also recorded in the foci register (Additional file 6) which was kept at the Regional VBDC Office. Both the malaria case register (Additional file 5) and foci register (Additional file 6) were also entered into the database and submitted electronically to the Central NMCP (Regional Malaria Assistant, personal communication on 10th Nov 2022).

### Completeness and timeliness of reactive surveillance and response activities

At the national level, case notification was done for 77.4% (2114/2732), 79.3% (3274/4131) and 34.5% (706/2047) of positive cases in 2019, 2020 and 2021, respectively. Among the positive cases, 75.5% (2062/2732), 76.8% (3171/4131) and 23.4% (479/2047) were investigated in 2019, 2020 and 2021, respectively [14] (Table 3). Therefore, overall national completion rate of case notification and case investigation over three years period were 68.4% (6094/8910) and 64.1% (5712/8910), respectively, although no data is available to assess the timeliness of the RASR activities. At the sub-national level, it was noted that either the data availability was limited or completeness of RASR activities was low (Table 3).

**Table 3:** Completeness of RASR activities at state and regional level in Myanmar in 2019–2021 [14]

State/Region	2019				2020				2021			
	Index malaria cases	Case notification n (%)	Case investigation n (%)	Foci investigation and response^a^	Index malaria cases	Case notification n (%)	Case investigation n (%)	Foci investigation and response^a^	Index malaria cases	Case notification n (%)	Case investigation n (%)	Foci investigation and response^a^
**National**	**2732**	**2114 (77.4)**	**2062 (75.5)**	**493**	**4131**	**3274 (79.3)**	**3171 (76.8)**	**592**	**2047**	**706 (34.5)**	**479 (23.4)**	**33**
Ayeyarwady	116	45 (38.8)	45 (38.8)	29	45	36 (80.0)	36 (80.0)	34	171	103 (60.2)	46 (26.9)	7
Bago	521	-Na-	370 (71.0)	62	356	-Na-	251 (70.5)	43	576	-Na-	87 (15.1)	3
Chin	-Na-	-Na-	-Na-	-Na-	-Na-	-Na-	-Na-	-Na-	-Na-	-Na-	-na-	-Na-
Kachin^b^	-Na-	-Na-	-Na-	-Na-	593	486 (82.0)	468 (78.9)	9	1275	179 (14.0)	179 (14.0)	0
Kayah^b^	-Na-	-Na-	-Na-	-Na-	102	88 (86.3)	93 (91.2)	10	-Na-	-Na-	-Na-	-Na-
Kayin^b^	-Na-	-Na-	-Na-	-Na-	62	-Na-	16 (25.8)	-na-	160	-Na-	7 (4.4)	-Na-
Magway	-Na-	-Na-	128 (Na)	72	-Na-	-Na-	105 (Na)	62	-Na-	-Na-	-Na-	-Na-
Mandalay	-Na-	-Na-	-Na-	-Na-	260	-Na-	152 (58.5)	43	116	-Na-	-Na-	-Na-
Mon	894	-Na-	271 (30.3)	77	1265	-Na-	485 (38.3)	31	-Na-	-Na-	-Na-	-Na-
Nay Pyi Taw Union Territory	55	-Na-	46 (83.6)	35	83	-Na-	39 (47.0)	37	40	-Na-	15 (37.5)	6
Rakhine^b^	1136	-Na-	-Na-	-Na-	758	257 (33.9)	168 (22.2)	51	1432	770 (53.8)	694 (48.5)	22
Sagaing^b^	-Na-	-Na-	-Na-	-Na-	75	-Na-	62 (82.7)	11	-Na-	-Na-	-Na-	-Na-
Shan (East)	100	-Na-	-Na-	-Na-	97	-Na-	-Na-	-Na-	20	-Na-	-Na-	-Na-
Shan (North)	-Na-	-Na-	-Na-	-Na-	1100	-Na-	619 (56.3)	95	891	-Na-	29 (3.3)	14
Shan (South)	-Na-	Na-	Na-	Na-	147	Na-	56 (38.1)	26	29	Na-	12 (41.4)	12
Tanintharyi	-Na-	Na-	Na-	Na-	8214	-Na-	Na-	Na-	8764	-Na-	Na-	Na-
Yangon	53	45 (84.9)	45 (84.9)	14	32	31 (96.9)	31 (96.9)	12	39	36 (92.3)	35 (89.7)	8

The bold values represent the national level figures, and they were bold to differentiate between the rest of the table which are sub-national level data

^a^ Foci investigation and response activities are not necessary for every positive case and hence percentages were not calculated

^b^Only data from elimination townships was described

The literature assessing the completeness and timeliness of RASR activities in Myanmar is also limited (Table 4). A study by Aye Mon Mon Kyaw et al. [15] assessed the completeness and timeliness of RASR activities based on the routinely collected programme data from Yangon, Bago-East and Mon State/Region from January to December 2016. It was documented that only 27.9% of the positive cases (268/959) were notified within 1 day, and 32.5% (312/959) were investigated within 7 days as per the national guidelines. It was also documented that 201 foci were investigated and received appropriate response measures within 7 days [15].

**Table 4:** Completeness and timeliness of RASR activities in Myanmar as reported in published literature

Author, year	Study area	Study timeframe	Total number of			
			Index malaria cases	Case notification conducted within 1 day n (%)	Case investigation conducted within 7 days n (%)	Foci investigation and response conducted within 7 days^a^
Aye Mon Mon Kyaw et al. 2018 [15]	Yangon, Bago-East, Mon	Jan-Dec 2016	959	268 (27.9)	312 (32.5)	201
San Kyawt Khine et al. 2019 [16]	Rakhine State (Toungup, Ramree, Munaung townships)	Apr 2018 to Mar 2019	171	-Na-	157 (91.8)	-Na-

^a^ Foci investigation and response activities were not necessary for every positive case and hence percentages were not calculated.

Conversely, another study by San Kyawt Khine et al. [16] reported that case investigation was done for 91.8% of the index malaria cases (157/171) in three townships of Rakhine State between April 2018 and March 2019 [16] (Table 2). These study areas were reported having a strong collaboration between NMCP and implementing partner, adequate human resource, financial support and other supplies [16].

### Enablers for reactive surveillance and response activities

One of the enablers for successful implementation of RASR activities was the good knowledge of frontline health workers on RASR activities. A study conducted in Myanmar described that 83% of the township level Basic Health Staff and VBDC staff knew the key RASR activities and 95% knew the activities to be conducted within one day [9]. Regional level NMCP staff also believed that knowledge of township level Basic Health Staff and VBDC staff of malaria elimination and RASR activities had improved over recent years as they became more reliable in performing RASR activities, especially malaria case notification and case investigation (Regional Malaria Assistant, personal communication on 10th Nov 2022).

Another enabler for RASR activities was the important coordination of Basic Health Staff in timely implementation of RASR activities especially foci response activities. Since local authorities in villages required continuous and comprehensive updates on the surveillance activities to provide strong administrative support, Basic Health Staff facilitated the process by linking VBDC team with local community and local authorities so that timely foci response activities could be conducted successfully [9].

### Barriers to reactive surveillance and response activities

One of the barriers for successful implementation of RASR activities in Myanmar was the shortage of human resources, especially in hard-to-reach settings [9]. Even though the MEFIM guidelines recommended establishing Malaria Elimination Management Teams at each elimination township to perform RASR activities within the respective township, this was not the case in most malaria elimination townships due to limited human resources at the township level. Therefore, the VBDC team from State/Regional level were required to be involved in all steps of the RASR (Regional Malaria Assistant, personal communication on 10th Nov 2022).

Barriers related to the surveillance system were also identified. Basic Health Staff reported that the major challenge for them in performing RASR activities was the real-time case notification and investigation [9]. This might be due to delays in reporting as a result of using the traditional paper-based reporting system in areas with limited mobile phone network services and internet coverage in Myanmar [9, 17]. Surveillance system barriers to timely implementation of RASR activities were further worsened by the lengthy and complex case investigation form and different reporting systems between Basic Health Staff and Integrated Community Malaria Volunteers, especially in those areas where the Malaria Case-based Reporting (MCBR) application (an older version of MCBRS application which only had the case management component but not the case and foci investigation component) was used [9]. Basic Health Staff also reported data inconsistencies between carbonless paper-based reporting system and MCBR electronic reporting system, resulting in data duplication or loss between these two systems as a challenge in implementing the RASR activities [9, 17].

In addition, other documented barriers for RASR included low level of community knowledge regarding health and wellbeing, inadequate supplies of malaria commodities, transportation difficulty, and inability to control the influx of migrant workers [9].

## Discussion

Overall, like other countries in the GMS, Myanmar has policy commitment, guidelines and standard operating procedures for implementation of each step of RASR strategies. However, Myanmar does not have specific guidelines for reactive case detection as in other GMS countries. In addition, Malaria Elimination Management Teams are yet to be established in most townships and, therefore, the Regional VBDC teams are responsible for all the foci investigation and response activities. This extra responsibility could present a burden for Regional VBDC teams in the long run since the team is also responsible for other vector-borne diseases such as dengue and lymphatic filariasis.

This literature review noted that completeness of RASR activities varied across different time periods and geographical areas. Completeness of RASR activities was found to be low in the earlier study conducted in 2016 [15] compared to the more recent one conducted in 2018–2019 [16]. This is likely because RASR activities were only introduced in Myanmar in 2016 and at that time there were no specific guidelines, standard operating procedures, or proper documentation for field implementation of these activities. Review of sub-national data also noted that completeness of RASR activities was low in the least developed states and regiona with high malaria transmission (e.g., Chin, Kachin, Kayah and Kayin) and those with human resource shortage in health sector (e.g., Sagaing and Tanintharyi) compared to developed and urbanised, and low malaria transmission states and regions (e.g., Yangon).

It was also noted that implementation of RASR activities in Myanmar is challenging due to several limitations in the health system. Implementation of RASR activities is an intensive work which needs collaborative effort from different factions of health staff, therefore inadequate human resources may put extra burden on the existing staff. Constraints of the national malaria surveillance system include lengthy and complex case investigation forms and by data discrepancies between paper-based and electronic reporting systems potentially jeopardize the validity of the data. Shortages in the supplies of malaria commodities including rapid diagnostic tests and antimalarial drugs are an additional barrier to effective RASR activities in Myanmar. Due diligence should be undertaken to address these health system related challenges in order to achieve the goal of malaria elimination by 2030.

Apart from health system limitations, other factors that undermined the RASR activities included low level of community knowledge regarding health and wellbeing, transportation difficulty, and inability to control the movement of migrant workers. Like other public health interventions, successful implementation of RASR activities requires community engagement and it is important that the community itself must define, believe in, and commit to malaria elimination strategies [18]. In addition, it is important to take timely response measures in order to interrupt the onward transmission of malaria, especially in the high-risk populations [19]. Transportation difficulties and undocumented migration patterns might cause delays in implementing appropriate and effective response activities and increase risk of onward transmission.

## Limitations

The number of studies assessing the completeness and timeliness of malaria RASR strategies in Myanmar is very limited and this literature review highlighted an important knowledge gap for malaria surveillance and responses essential in the Myanmar Malaria Elimination Programme. In addition, most of the published and grey literature primarily assessed the completeness of RASR activities and not the timeliness.

## Conclusion and recommendations

- According to the MEFIM guidelines, foci investigation and response activities are supposed to be conducted by the township or district level Malaria Elimination Management Team, however, Malaria Elimination Management Teams are yet to be established in most areas. Establishment of Malaria Elimination Management Team with a dedicated team leader is necessary in all areas aiming to eliminate malaria in order to implement RASR activities effectively.
- Completeness of RASR activities was found to be high in areas with adequate resources and RASR strategies need to be tailored to the existing health and surveillance system as well as the local context of the states and regions.
- Collaboration between the NMCP and its implementing partners needs to be strengthened so that the gaps in the township Malaria Elimination Management Team can be fulfilled by implementing partners.
- It was noted that case investigation form was lengthy and complex. A simplified version of case investigation form which is more user-friendly for field level staff should be developed and deployed.
- There is limited data to assess the timeliness and effectiveness of RASR activities in Myanmar. A comprehensive national level assessment of RASR strategies should be conducted to optimise the RASR and ultimately accelerate towards malaria elimination by 2030.
- Health system strengthening and policy commitment for malaria elimination should be undertaken.
- Strategy and standard operating procedures for reactive case detection should be developed.

## Supplementary information

**Additional file 1: **Malaria positive case notification form.

**Additional file 2: **Modified case investigation form.

**Additional file 3: **Case classification form.

**Additional file 4: **Modified focus investigation form.

**Additional file 5: **Translated version of malaria positive case register.

**Additional file 6: ** Foci register.

## Data Availability

Not applicable.

## Abbreviations

GMS: Greater Mekong Sub-region
ITN: Insecticide treated net
IRS: Indoor residual spraying
LLIN: Long-lasting insecticidal net
MEFIM: Malaria elimination field implementation manual
NMCP: National Malaria Control Programme
RASR: Reactive surveillance and response
VBDC: Vector borne disease control (Programme)
WHO: World Health Organization
