# Supplementary material for: Reactive surveillance and response strategies for malaria elimination in Myanmar: a literature review
Source: Malar J. 2023 Apr 27;22:140. doi: 10.1186/s12936-023-04567-6 (PMC10141915; doi:10.1186/s12936-023-04567-6)
Supplement: Supplementary file 1 — Additional file 1: Malaria positive case notification form. [file 12936_2023_4567_MOESM1_ESM.docx]

**Additional file 1: Malaria positive case notification form (Source: Malaria Elimination Field Implementation Manual version 1.00)**

| Immediate notification of malaria positive case to the Township Malaria Elimination Management Team | |
| --- | --- |
| Details of notifier who reports the positive malaria case | |
| Name and designation |  |
| Village name |  |
| Contact phone number |  |
| Date of notification |  |
| Means of notification | \| Mobile  Phone \| SMS message \| others \| \| --- \| --- \| --- \| |
| Notified malaria case | |
| Positive malaria species |  |
| Date of onset of fever |  |
| Date of testing (diagnosis) |  |
| Clinical status of malaria case | \| Uncomplicated malaria \| Severe complicated malaria \| Death (Date) \| \| --- \| --- \| --- \| |
| Date and place of travelling during the last one month before the onset of fever | Name of place of travelling –  Date of travelling From------------------- to ------------------ |
| Detail facts about positive case | |
| Name of positive case |  |
| Age and Sex | \| Age \| Yr/ Months \| Sex \| Male \| Female \| \| --- \| --- \| --- \| --- \| --- \| |
| Patient's contact phone # |  |
| Name of head of family members |  |
| Permanent address |  |
| Positive case present address |  |
| *VBDC, Myanmar, January-2020* | |
