# Supplementary material for: Reactive surveillance and response strategies for malaria elimination in Myanmar: a literature review
Source: Malar J. 2023 Apr 27;22:140. doi: 10.1186/s12936-023-04567-6 (PMC10141915; doi:10.1186/s12936-023-04567-6)
Supplement: Supplementary file 2 — Additional file 2: Modified case investigation form. [file 12936_2023_4567_MOESM2_ESM.docx]

**Additional file 2: Modified case investigation form (Source: Malaria Elimination Field Implementation Manual version 1.00)**

Section (A) 1. (Patient's general information)

| 1. **Patient's Particulars**   Patient’s name_________________ Positive case ID no. ______________ Carbonless Reg ***(Sr no/mth/yr)*** ________  Age______ year Male/Female _______ Occupation ________________ Father’s name ________________  ***Permanent address***  House No.______________, Street___________________, Village/Ward_______________  SHU/RHC ___________________________, Sub-RHC____________________  Township _________________, State/ Region_____________________  Contact phone number_____________________ *(PCO)* ____________________  GPS Location (Decimal Degree) **Latitude**___________ **Longitude**____________ (E.g ##.#####) |
| --- |

| Place of blood testing ***(ward/village and Township)*** ­­­­____________________________________________ | |
| --- | --- |
| Sub-RHC___________________________ | SHU/RHC_______________________ |
| Township___________________________ | State/Region_________________________ |
| P. code  ________________________________ | GPS Location (Decimal Degree)  **Latitude**___________ **Longitude**____________ (E.g ##.#####) |
| Date of onset of fever _____________________ |  |
| **Date of blood testing**_________________________ | **Positive species**____________________________ |
| **Date of Notification**______________________ | **Date of Case Investigation**____________________ |
| Place where case investigation was conducted ***(ward/village and Township)*** ­­­­_______________________________________________ | |

Section (B) 2. and 3. (Classification of positive case)

| 2.1 Any blood transfusion during the last 3 months Yes No Date of blood transfusion _____________ |
| --- |
| 2.2 Any past history of malaria within the last three years ***(Not including the previous 30 days. (It is separately interview in no.3)***  Before this current malaria attack, was there any previous malaria attack within the last three years?  No ***(If “No”, skip the remaining 2.2 and continue to 3.)***  Yes when _______/_______ *(Month/Year)*  Any blood test during that time Yes No Not remember  If anti-malaria drugs taken, how many days taken (Estimated) ________________________  Taken completely according to instruction or not Yes No |

**3. Detail Travelling History** – overnight stay by the patient during the previous 30 days

No ***(If "No", skip 3-1 and 3-2; continue to interview 5) …… This case may be Locally Contracted Malaria case.***

Yes If "Yes", please fill the following tables 3-1 and 3-2. (To be filled all places where the patient stayed overnight). Correlate these two tables and find out where the patient was present during the incubation period of the malaria parasites. Then differentiate between locally contracted malaria cases and imported cases.

3-1. Travelling history during past 30 days by positive case

| No. | Place  **(Township/ward/ village/worksite)** | Township | State/Region | Period of visit  **(Day/Month/Year)** | | Any night stay in this place  **(Yes/No)** |
| --- | --- | --- | --- | --- | --- | --- |
|  |  |  |  | From | To |  |
| 1 |  |  |  |  |  |  |
| 2 |  |  |  |  |  |  |
| 3 |  |  |  |  |  |  |
| 4 |  |  |  |  |  |  |
| 5 |  |  |  |  |  |  |

3-2. Identify where the patient was during the incubation period?

| Day | **Date of onset of fever**  Day 1 | Day 2 | Day 3 | Day 4 | Day 5 | Day 6 | Day 7 | Day 8 | To fill the date backwards starting from **"Date of onset of fever."** |
| --- | --- | --- | --- | --- | --- | --- | --- | --- | --- |
| Date |  |  |  |  |  |  |  |  |  |
| Day | Day 9  (*Pf*) | Day 10  (*Pf*) | Day 11  (*Pf*) | Day 12  (*Pf/Pv*) | Day 13  (*Pf/Pv*) | Day 14  (*Pf/Pv*) | Day 15  (*Pv*) | Day 16  (*Pv*) | Day 17  (*Pv*) |
| Date |  |  |  |  |  |  |  | | |
| Pf incubation period |  |  |  |  |  |  | Identify where the patient was? | | |
| Pv incubation period | Identify where the patient was? | | |  |  |  |  |  |  |
| ***Incubation period for Pf –9^th^ to 14^th^ day; Pv 12^th^ to 17^th^ day. Identify where the patient was during the incubation period.***  ***Remarks – Apart from travelling days, the patient was at the village in remaining days.*** | | | | | | | | | |

***Decision –*** (1) Parasite species – Pf or Pv or mixed

(2) During the incubation period of particular parasite species, the patient is present in (name of place) …………………………… ……………………………………… from (Date)……………………………..to (Date)…………………………………..So, the case is locally contracted malaria case / imported case.

Remarks – If the case is **locally contracted malaria case**, **continue the remaining all sections** in serial.

- If the case is **imported case, skip section C and continue the remaining sections.**

Section (C) 4. Further classification of locally contracted malaria case to indigenous or introduced

If the patient was **locally contracted malaria case** and source of infection was imported case, the case was classified as **introduced case.** If the source of infection was indigenous, the case was also classified as **indigenous**.

| 1. Differentiation of **Introduced case** and **Indigenous case** (not to ask the patient, review positive case register/ case investigation form by MEMT)  \|  \| Yes \| No \| \| --- \| --- \| --- \| \| (a) \|  \|  \| \| (b) \|  \|  \| \| (c) \|  \|  \| \| (d) \|  \|  \|  1. At least three weeks period from the onset of fever, there was no travelling history of positive (locally contracted malaria) case 2. Around the last three weeks before the first attack of the current patient, there was/were positive case/cases in that area/village. 3. Among those positive cases, there was/were Imported case(s) included according to case investigation 4. Species of **Imported case** is the same with the species of the current patient.   *If all (a,b,c,d) are "Yes", it is* ***Introduced case****.*  *If at least one result was "No", it is* ***Indigenous case****.*  **Introduced case Indigenous case**  **The above information must be reviewed in the Positive Case Register and confirmed by Township Supervisor.** |
| --- | --- | --- | --- | --- | --- | --- | --- | --- | --- | --- | --- | --- | --- | --- | --- |

Section (D) 5 to 7. Determination of the onward transmission

| **5. During this current attack (before completion of Rx), any places where patient stayed overnight. (Please fill the travelling period in the table according to chronological order)** | | | |
| --- | --- | --- | --- |
| Place  **(worksite/village, Township and State/Region)** | Starting date of travelling | End date of travelling | Slept under bed net during travelling  Yes/No |
|  |  |  |  |
|  |  |  |  |
|  |  |  |  |
|  |  |  |  |

| **6. Bed net utilization status of patient**  Total number of household member- (………….….) | | |
| --- | --- | --- |
| **Ask to the positive patient** | | |
| What type of bed nets? | Ordinary net | LLIN |
| Sleep under bed net during the period from onset of fever to blood testing. **(Yes/No)** |  |  |
| Sleep under bed net since after blood testing up till now. **(Yes/No)** |  |  |
| **Ask the household members** | | |
| Number of household members who always sleep under bed net |  |  |
| Number of HH members who did not sleep under bed nets (**during the patient’s febrile episode and while receiving treatment**) |  |  |
| Please mention the reasons why not slept under bed net? |  |  |

| **7. Any Breeding Place near-by patient’s resident house?** *(To be identified by interviewer)* | | | |
| --- | --- | --- | --- |
| No | Type of breeding place – wells (Mon & Kayin),  *Slow running stream, pools/ponds,rice-field, fish/prawn farms, irrigation cannel, hand-scoop* wells, tyre track, animal hoofs,lagoon (creek), *connection of salanity and fresh water, rocky depression, Others(specify)* | Number of larvae  1-Few  2-Moderate  3-Abundant | It is near/surrounding of patient's house or not.  Yes/No |
| 1. |  |  |  |
| 2. |  |  |  |
| 3. |  |  |  |

Section (E) 8. Reactive case detection around the house of index case

Use Table from Foci Investigation Team. (Foci Investigation Team collects this information)
