# Supplementary material for: Reactive surveillance and response strategies for malaria elimination in Myanmar: a literature review
Source: Malar J. 2023 Apr 27;22:140. doi: 10.1186/s12936-023-04567-6 (PMC10141915; doi:10.1186/s12936-023-04567-6)
Supplement: Supplementary file 3 — Additional file 3: Case classification form. [file 12936_2023_4567_MOESM3_ESM.docx]

**Additional file 3: Case classification form (Source: Malaria Elimination Field Implementation Manual version 1.00)**

| 10. Patient’s name and age_____________________၊ _______year  Ward/Village/Worksite_____________________________  Date of onset of fever______________________၊ Species___________  Any travelling history for 30 days before this current attack. Yes No | | |
| --- | --- | --- |
| **Case Classification (Please choose only one classification from below) if it is imported case, please choose one from a,b,c.** | | |
| 10.1 | **Indigenous** |  |
| 10.2 | **Introduced** |  |
| 10.3 | **Imported**  (a) **Outside the village but within the same township**  Name of contracted village…………………၊ forest………………….၊ worksite……………(Please describe) *  (b) **Outside the township but within the same State/Region**  Name of contracted Township…………………….၊ village/forest/worksite………………(Please describe)*    (c) **Outside the State/Region but within the Country –Name of State/Region ………………………….**  Name of contracted Township……………………၊ village/forest/worksite………………(Please describe)* | Describe about  POSOI----- |
| 10.4 | **Relapse/ Recrudescence** |  |
| 10.5 | **Induced** |  |
| 10.6 | **Cryptic** |  |

* Please describe about the place of source of infection (POSOI)

Name of investigator Name of Supervisor

Designation Designation

Signature Signature
