# Supplementary material for: Reactive surveillance and response strategies for malaria elimination in Myanmar: a literature review
Source: Malar J. 2023 Apr 27;22:140. doi: 10.1186/s12936-023-04567-6 (PMC10141915; doi:10.1186/s12936-023-04567-6)
Supplement: Supplementary file 4 — Additional file 4: Modified focus investigation form. [file 12936_2023_4567_MOESM4_ESM.docx]

**Additional file 4: Modified focus investigation form (Source: Malaria Elimination Field Implementation Manual version 1.00)**

**1. General information on focus**

Date of this report: _____________Date of focus Investigation _____________ Malaria Focus ID:_______ State/Region: ____________ Township: _____________ Village/Worksite:_________________________

RHC ____________________ Sub-centre: ________________ Village ID: ____________________________

GPS LOCATION (Latitude________________ Longitude_________________)

INFORMATION FROM PRIMARY CASE INVESTIGATIONs

Name of malaria positive case___________________________ Positive case ID ____________________

Classification of malaria case as to origin of infection: ________________________________________

**1.1** Geographical **map** of focus: insert as Figure 13

**1.2** Receptivity **status of the foci area:** Rural Urban Altitude: ……………

Please transform the collected data using “Table 12: Data collection form for Receptivity status of foci” into narrative form __________________________________________________________________________________________________________________________________________________________________________________

**1.3** Vulnerability **status of the foci area:**

1.3.1.*Explain proximity to endemic areas across region/ international borders, displaced people, refugees)*: _______________________________________________________________________________

1.3.2. Please transform the collected data using *Table 13: Data collection form for vulnerability status of “Resident villagers” AND Table 14: Vulnerability status of incoming “Migrant villagers migrated to Investigated village”* into narrative form _____________________________________________________

**1.4 Assessment on health care services (Malaria surveillance system)**

Please transform the collected data using *Table 15: Data collection form for status of health care services related to malaria (Part A), AND “Table 16: Data collection form for status of health care services related to malaria (Part B)*; into narrative form ________________________________________

**1.5. Assessment on socio-behaviour characteristics of the population in assessment village**

Mention about occupational factors, some traditional spiritual festivals, etc., if related to occurrence of malaria, sleeping habits (sleep in the farm hut without wall and not using LLIN), treatment seeking behaviour, night-time economic activities like hunting. Also mention positive behaviour like using repellent and knowledge, attitude, practice on malaria including outdoor staying before bedtime. Also transform data from Table 17: Sociodemographic data and LLIN ownership of foci area AND Table 18: (Continued from Table 16) Sociodemographic data, LLIN ownership of foci area and treatment seeking behaviour in foci investigation to narrative information.

**1.6 Characteristics of malaria vectors within the focus (if previous data were available).**

**(**Entomological assessment should take part in problem foci area where type of foci is not clear)

**2. Past history of malaria interventions in focus village**

**2.1 LLIN distribution campaigns** within the focus in the past three years: YES NO; If YES:

| Year of distribution | LLINs distributed  (A) | Target  population  (B) | Coverage:  (A) x 2  (B) | Remarks |
| --- | --- | --- | --- | --- |
|  |  |  |  |  |
|  |  |  |  |  |
|  |  |  |  |  |

Suspected reasons for possible reduced effectiveness of LLINs (e.g. frequent washing, smoke deposit, use of conventional nets, outdoor human behaviour/work, sleeping habits): ……………….…………………………….…………………….…………………………….……

**2.2 IRS activities** within the focus in the past three years: YES NO; If YES:

| Date of IRS | Insecticide used | Target houses | Houses sprayed | Coverage | Remarks |
| --- | --- | --- | --- | --- | --- |
|  |  |  |  |  |  |
|  |  |  |  |  |  |

**3. Classification of the focus**

**Relation of the focus to the index case** that prompted focus investigation:

- Classification of the index case (e.g. indigenous, imported): _______________________________________

**3.1 Classification of focus**

**- Before detection of this positive case**

Active Residual non-active Cleared Other: _________________

**- After detection and classification of this case**

Active Residual non-active Cleared Other: _________________

Parasite species: (put positive numbers)

*P. falciparum P. vivax P. malariae*

Mixed, specify: ……… Other, specify:

**3.2. Patient’s resident village and Foci classification based on place of source of infection (POSOI)**

- Positive case’s resident village name ___________________

- Foci classification of resident village _________________

- If source of infection is not from the resident village, specify the place explicitly from where he/she got malaria ______________________________________________________________________________

- Name of place of source of infection____________________ Foci classification of POSOI ____________

**4. Response (Summary)**

Measures implemented, to clear infections, stop transmission within the focus, and prevent possible onward spread of the infections from the focus. All detail activities should be attached.

**4.1. Regular key response activities**

| **Measures taken** | **Remarks** |
| --- | --- |
| Case Detection  (specify PCD/ACD/ RACD/PACD) | Date: _____/_____/_____ No. tested: _____ No. positives: _____________  Total positives treated_____________  Parasite species: ­­­­­­­­­­­­­­­­_________________________________________________ |
| Larval source reduction | Date: _____/_____/_____ Places treated: ____________________________  Method used: physical/ chemical/ biological (If “Chemical method”, name the insecticide_____________________________________________ |
| LLIN distribution | Date: _____/_____/_____ No. of LLINs distributed: ________________________ |
| Indoor Residual Spraying | Date: _____/_____/_____No. of Houses sprayed: ____________________________  Insecticide used: _________________________________________________ |
| Other vector control measures |  |
| Strengthening Surveillance |  |
| Behavioural Change Communication |  |
| Other measures |  |

**4.2.** **Specific measures based on CIFI findings *(If you need more rows, please use copy table)***

| No. | Findings based on data collection | Proposed response activities to be implemented on Day 3, based on CIFI findings | Actual implementation. (Most of the activities need data) |
| --- | --- | --- | --- |
| 1 | Receptivity (Weaknesses) |  |  |
|  |  |  |  |
|  |  |  |  |
|  |  |  |  |
|  |  |  |  |
|  |  |  |  |
| 2 | Vulnerability (Weaknesses) |  |  |
|  |  |  |  |
|  |  |  |  |
|  |  |  |  |
|  |  |  |  |
|  |  |  |  |
| 3 | Health care services (Weaknesses) |  |  |
|  |  |  |  |
|  |  |  |  |
|  |  |  |  |
|  |  |  |  |
|  |  |  |  |
| 4 | Behaviour of the population (Weaknesses) |  |  |
|  |  |  |  |
|  |  |  |  |
|  |  |  |  |
|  |  |  |  |
|  |  |  |  |
| 5 | Mention the strengths based on above 4 factors | | |
|  |  | | |
|  |  | | |
|  |  | | |
|  |  | | |
|  |  | | |

Date :_____/_____/_____ Date : _____/_____/_____

Name of responsible officer: Counter-checked by (Name)

________________________ __________________________________

Title of responsible officer Title of responsible officer Malaria RO / TL

___________________________ ________________________________________

Signature of responsible officer Signature of Malaria RO/ TL

___________________________ ________________________________________
