# Supplementary figures and images for: Reactive surveillance and response strategies for malaria elimination in Myanmar: a literature review
Source: Malar J. 2023 Apr 27;22:140. doi: 10.1186/s12936-023-04567-6 (PMC10141915; doi:10.1186/s12936-023-04567-6)

**Additional file 5: Translated version of malaria positive case register**


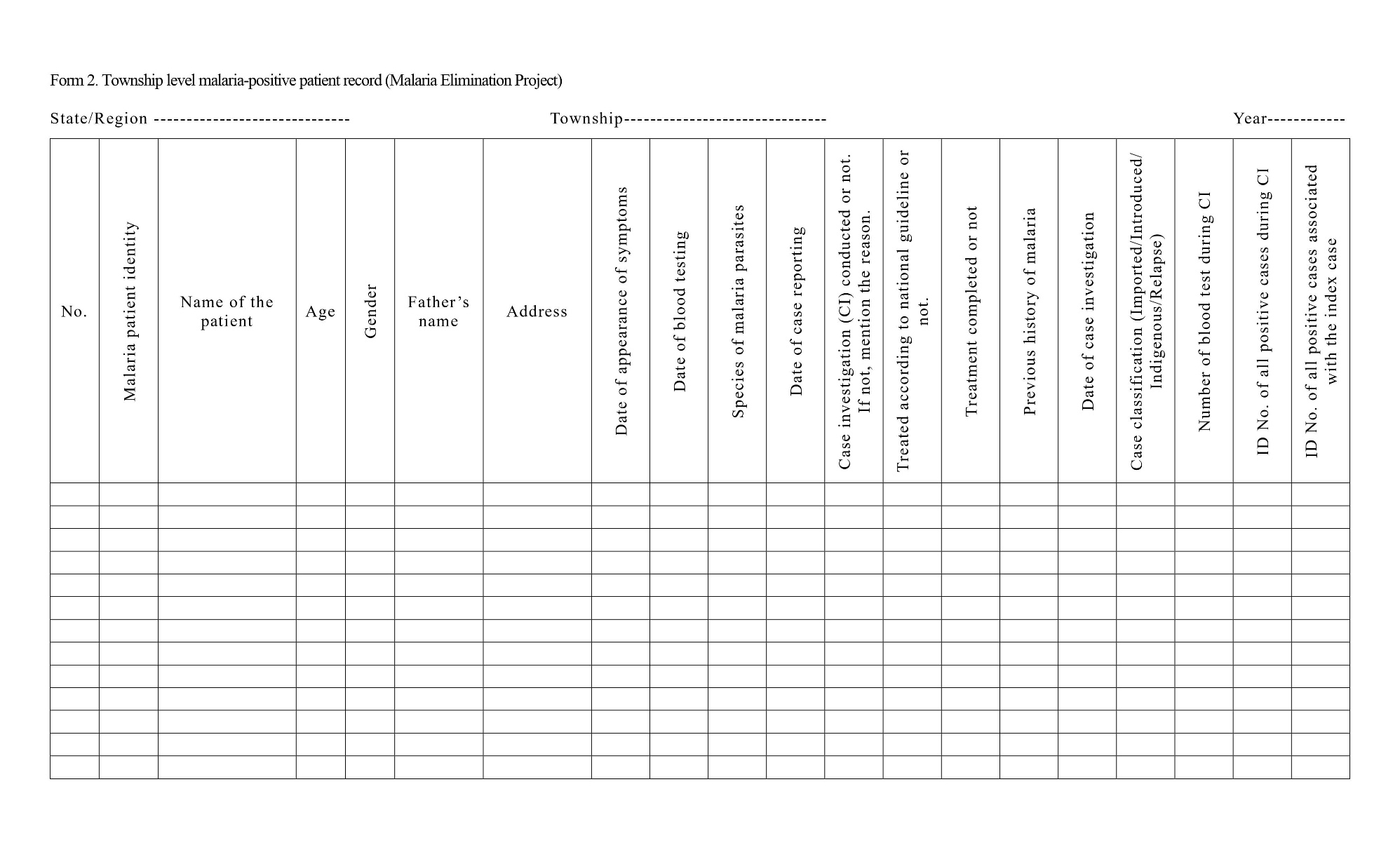

Supplement: Supplementary file 5 — Additional file 5: Translated version of malaria positive case register. [file 12936_2023_4567_MOESM5_ESM.docx]

**Additional file 6: Foci register**

**
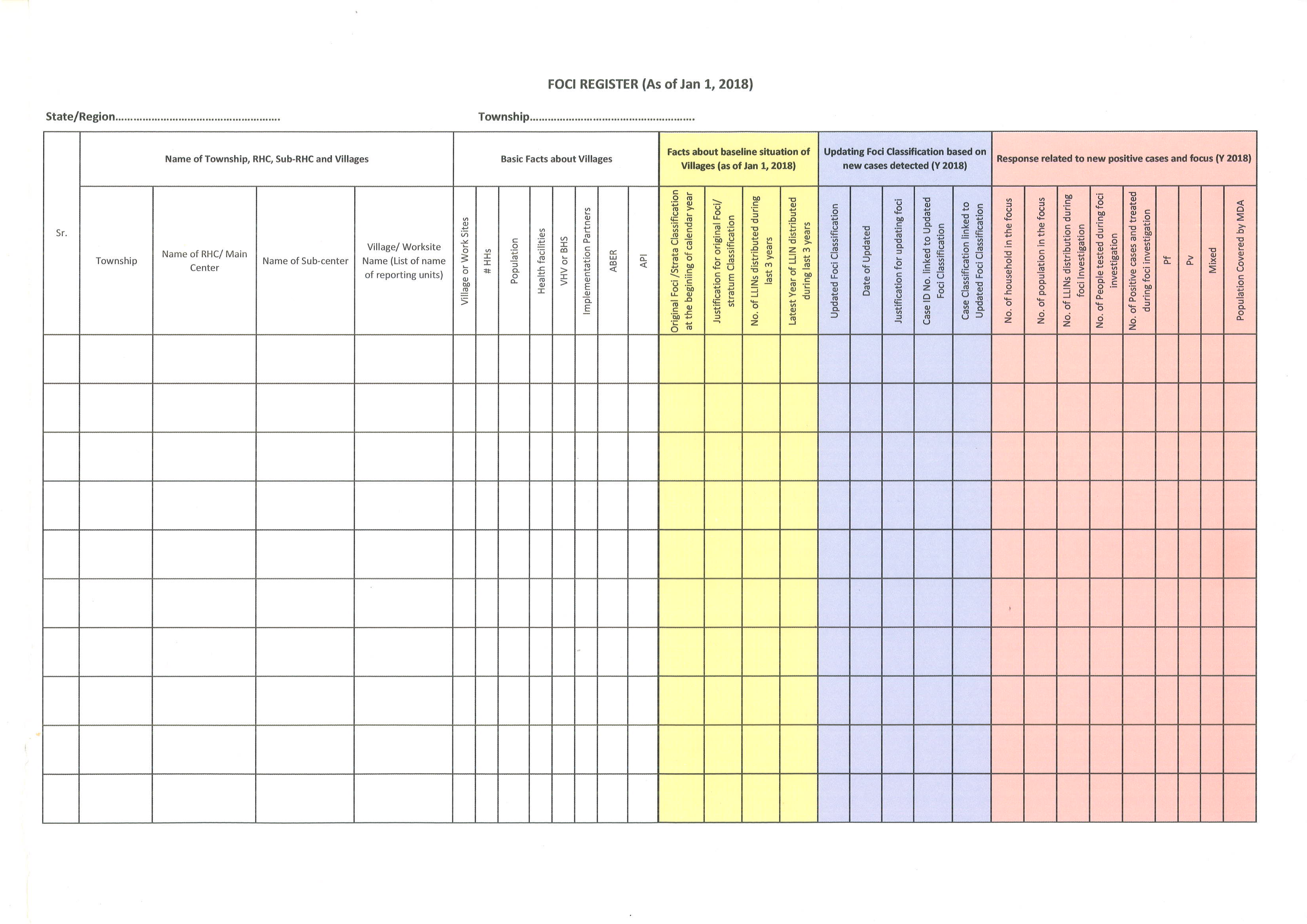
**

Supplement: Supplementary file 6 — Additional file 6: Foci register. [file 12936_2023_4567_MOESM6_ESM.docx]
